# Parturitions, menopause and other physiological stressors are recorded in dental cementum microstructure

**DOI:** 10.1038/s41598-020-62177-7

**Published:** 2020-03-25

**Authors:** Paola Cerrito, Shara E. Bailey, Bin Hu, Timothy G. Bromage

**Affiliations:** 1 Department of Anthropology, New York University, New York, USA; 2 New York Consortium in Evolutionary Primatology, New York, USA; 3 Department of Molecular Pathobiology, New York University College of Dentistry, New York, USA; 4 Department of Biomaterials, New York University College of Dentistry, New York, USA

**Keywords:** Biological anthropology, Menopause, Cementum

## Abstract

The life history pattern of recent humans is uniquely derived in many of its aspects including an extended post-reproductive lifespan combined with short interbirth intervals. A number of theories have been proposed to explain the evolution of this unusual pattern. However most have been difficult to test due to the fragmentary nature of the hominin fossil record and the lack of methods capable of inferring such later life history events. In search of a method we tested the hypothesis that the physiologically impactful events of parturition and menopause are recorded in dental cementum microstructure. We performed histomorphological analyses of 47 teeth from 15 individuals with known life history events and were able to detect reproductive events and menopause in all females. Furthermore, we found that other stressful events such as systemic illnesses and incarceration are also detectable. Finally, through the development of a novel analytical method we were able to time all such events with high accuracy (R-squared = 0.92).

## Introduction

The life history pattern of recent humans is uniquely derived in many of its aspects^1^. Unlike other large-bodied hominids we have a long gestation period but give birth to extremely altricial offspring; interbirth intervals are short and weaning occurs relatively early but childhood is long; and human females become infertile long before the end of their lifespan. Humans uniquely deviate from the great ape trend in that some traits of our life history appear to have accelerated (shortened interbirth intervals) and others are entirely new^2^ (the concept of a childhood and a post reproductive lifespan).

The evolution of our derived life history pattern has been difficult to investigate, due to the fragmentary nature of the hominin fossil record^2–4^ and to the lack of methods capable of detecting and timing later life history events. Most of our current knowledge regarding the evolution of hominin life history comes from the study of dental remains. Teeth are formed by incremental tissues, so that information regarding the pattern and rate of dental development, as well as certain life history parameters (age at weaning) are faithfully preserved. Initially reported by Adolf Schultz^5,6^, anthropologists have known for several decades that there is a strong correlation between dental development and life history parameters across a large number of primate species^7,8^.

With advances in histological methods, odontochronology and skeletochronology (the study of growth layers in teeth and bones, respectively) have been increasingly applied to the study of life history evolution. Mineralized tissues such as bone, enamel, dentine and cementum preserve incremental structures that are related to repeated physiological cycles^9^ and can be interpreted in a similar way as tree growth rings^10^. As aberrant growth lines in these tissues derive from physiological stressors that impact normal matrix formation, they are correlated to events such as birth, weaning, malnutrition, disease, climate and lifestyle changes^11–15^. Enamel and dentine have received the most attention in the anthropological literature; however, their primary secretions cease when tooth formation is completed. Thus, while they are useful for inferring age at weaning and gestation length, they are inadequate for investigating later life history events such as parturitions (with the exception of early life pregnancies^16^) and cessation of fertility. Therefore, our knowledge of the timing of post-weaning^17,18^ life history milestones in hominins is based on information (such as inferred body mass and brain size) that has been shown to either influence life history scheduling or to be correlated with other life history variables^19^. Currently, no direct methods are available to infer age at reproductive events (RE) and length of post-reproductive lifespan (PRLS) from mineralized tissues. This is because enamel and dentine formation is usually completed before these events occur, and because the remodeling activity of bone complicates the reconstruction of an individual’s chronology. These limitations impede our ability to answer the following questions: i) when did a PRLS first emerge?; and ii) does the evolution of a PRLS coincide with the reduction of the interbirth intervals (as posited by the grandmother hypothesis^20^)? To address these outstanding questions we endeavored to develop a new method and turn to the analysis of dental cementum, the only incrementally growing mineralized tissue that is deposited throughout an individual’s life, but is not subject to remodeling^21^.

**Figure 1:**
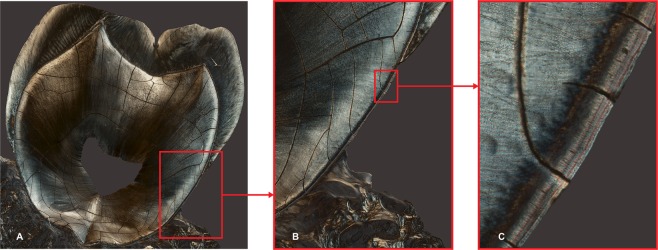
Montage on the X and Y planes of micrographs obtained in polarized light (PL) of a longitudinal section of the maxillary second molar of specimen M15-02. From left to right: whole tooth section (**A**), magnification of A (**B**), magnification of B (**C**). Magnification at acquisition is 1.3 micrometers per pixel, images A and B where compressed for publication as file size was too large, image C is in the original resolution. In image C: to the left is the dentine, covered by the layers of cementum; three distinct cementum bands are visible.

Teeth are made of an enamel cap covering a dentine core, which contains a pulp chamber where nerves, blood vessels and connective tissues are contained. The tooth’s root is covered by cementum (Fig. 1), which is secreted and mineralized in incremental layers. As recently reviewed in Dean *et al*.^22^ cementum is part of the periodontium, which functions to attach and protect the tooth within the bony dental alveolus^23^. Collagen fiber bundles, extending between the alveolar bone and the root surface, form the periodontal ligament (PDL). Cementum is secreted and mineralized in incremental layers over the root surface. Extrinsic collagen fibers of the PDL remain embedded within the cementum, giving rise to Sharpey’s fibers. The orientation of these fibers reflects the tensile loads experienced by the tooth, and they run approximately perpendicular to the cementum layers. Research has shown that root surfaces not covered by cementum are subject to resorption. Hence a fundamental function of cementum is to protect the root^24^. As new layers of cementum are periodically added, they serve to limit the downgrowth of gingival epithelium, which would induce deterioration of PDL ultimately leading to tooth loss^25^. Although both the alveolar bone and the PDL are subject to remodeling throughout life, the cementum is not^26^. Substances capable of regulating the growth of gingival fibroblasts may be released when needed and induce PDL regeneration^27^.

The majority of the inorganic mass of cementum is to be found in hydroxyapatite (HA) crystals [Ca_10_(PO_4_)_6_(OH)_2_], and constitutes approximately 65% of its wet weight. The HA crystals measure approximately 55 nm in width and 8 nm in thickness^28^. The composition of cementum varies histologically along the root surface: in the apical third Sharpey’s fibers cover only 40–60% of the cementum surface, whereas in the cervical two-thirds they may cover up to 100% of the cementum surface^22^. Intrinsic collagen fibers, which are secreted by cementoblasts and run parallel to the root surface, are present in the gaps between the large Sharpey’s fibers^29^. Intrinsic fiber cementum is a very plastic tissue^30^: the cementum adjacent to the root apex serves mostly a compensatory function against tooth movement and is secreted only after occlusion is achieved^31^. Cementoblasts found in the apical region are multipolar and have a higher secretion rate potential than those in the cervical region. These two factors result in thicker apical cementum and in cementoblasts that remain embedded within, giving rise to cellular cementum^32^. This contrasts with the cervical acellular extrinsic fiber cementum (AEFC)^21^. Incremental bands are visible in both cellular (apical) and acellular (cervical) cementum, although the two regions are very different. There are developmental differences between acellular and cellular cementum^33^.

The ontogenetic regulation of acellular cementum is obtained by cementoblasts modulating local levels of inorganic pyrophosphate (a physiological inhibitor of hydroxyapatite mineral precipitation) thus directing mineral apposition^34^. Additional evidence^28^ suggests that the two types of cementum (cellular and acellular) may be formed by populations of cementocytes having diverse origins. AEFC is found mainly in the cervical and middle thirds of the root. Its periodic deposition has been extensively noted and used for the purposes of age^31,35–38^ and season^39–41^ at death estimation in a variety of mammals, including humans. The biological bases of the layered appearance of cementum, which has been argued to present a pair of one light and one dark band per year, is still debated^29,31,42,43^. A number of validation studies^44–47^ using known-age human samples have achieved mixed results in accurately estimating age through counts of tooth cementum annulations (TCA). AEFC, unlike cellular cementum which dynamically responds to extrinsic factors such as mechanical load^21,32^, presents a slower but relatively constant growth rate^42,48,49^.

It is well established that, due to the important role that hard tissues play in the maintenance of organismal mineral homeostasis^50,51^, physiologically challenging events affect tissue mineralization. The skeleton is a dynamic organ involved in both endocrine regulation and global mineral homeostasis^52^. Physiologically demanding events such as pregnancy and lactation have multifold effects on the skeleton, including, but not limited to: upregulation of intestinal calcium absorption^53^, decrease of bone density^51,54^ and increased risk of maternal osteoporosis^55^. Across animal orders, the skeleton serves the purpose of mineral storage for the development of the fetal skeleton, and also of the egg in oviparous species^56,57^. The histological signature of birth and weaning in tooth enamel and dentine is well established in the literature^9,58^. The histological signature of pregnancy and lactation in dental cementum has been detected in a number of mammalian species^37,59–62^. These studies report that physiologically demanding events create a narrower cementum growth layer as a consequence of a slower progression of the mineralization front of cementoblasts. Because these histological signatures correlate with the year in which the stressful events occur, researchers have inferred age at parturition by measuring the thickness of each of the cementum annuli, with the thinner bands corresponding to birth year(s). These results are consistent with our current knowledge of the biological basis of seasonal alterations between dark and light cementum layers: the darker and thinner bands are thought to correspond to the physiologically stressful winter season, during which the cementum may become hyper-mineralized due to a slower advancement of the mineralization front^31^.

The physiological changes occurring in human females related to the cessation of fertility are also well known (e.g. decrease of estrogen and progesterone, increase in follicle-stimulating hormone and luteinizing hormone)^51^, and so are their effects on metabolism (e.g. decrease in energy expenditure and increase in adiposity)^55^ and hard tissue mineralization (e.g. bone loss)^54^. Bone composition and quality change drastically during female PRLS, across ethnicities^28^, as indicated by the high incidence of osteoporosis in post-reproductive females^63^. Based on this current body of knowledge, it is reasonable to predict that these physiological events also impact the mineralization of dental cementum. Indeed, previous research on a variety of mammals shows that events impacting the physiology of an organism, such as a change in diet^64^, exposure to exceptional cold^65^ and renal diseases^38^ affect the histomorphology of cementum, and likely also its elemental composition. Humans are one of only five mammalian species^66^ that experience a PRLS (and the only terrestrial one), hence comparative data from other organisms are unavailable.

Based on previous research, we predict that changes in organismal mineral homeostasis determine changes in the microstructure of cementum, which in turn can be detected by changes in the birefringence of the material when investigated by polarized light microscopy. We further predict that, since AEFC secretion rate is roughly constant^42,48,49^ throughout life, it should be possible to time significant physiological events (corresponding to changes in refractive index) occurring during tissue formation, by establishing proportional relationships between total cementum thickness and thickness of consecutive bands.

The aim of this study is to identify markers of parturitions, cessation of fertility and other physiological stressors in human dental cementum. We developed and tested a novel analytical method to infer RE per unit of time, age at menopause and PRLS from the analysis of the dental cementum. We applied this method to 47 contemporary *H. sapiens* teeth with known life history variables; and we tested the following hypotheses: (i) physiologically stressful events are recorded in cementum microstructure; and (ii) all teeth of an individual carry the same histomorphological signal in relation to stressors. Ultimately, we aim to provide methods capable of addressing some of the outstanding questions in human evolution regarding our peculiar life-history pattern. Because it has been already established that cementum can preserve well in the fossil record^22,67,68^, this study represents an important first step towards that goal.

## Results

### Age at physiological stressor estimation

**Figure 2:**
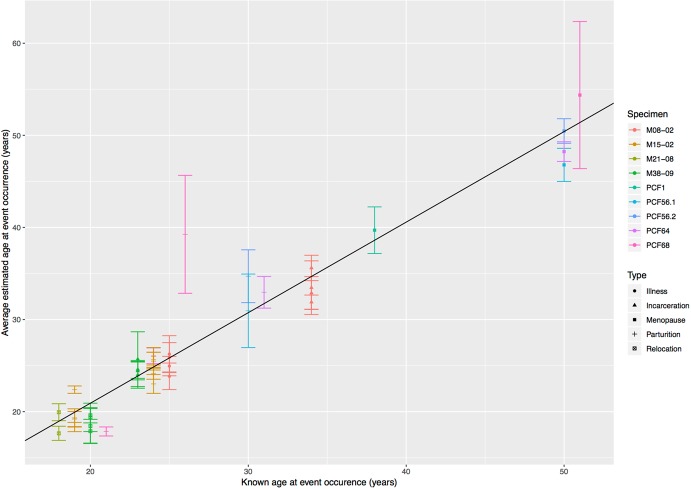
Bivariate plot showing the positive linear relationship between real and estimated age at event occurrence. Whisker plot shows mean ± 1 SD. The equation of the regression line is y = 0.94× +1.59 and the R-squared is 0.92. Individuals are color-coded, while the symbol indicates the type of event. Data used to generate this plot are reported in Supplementary Table 1.

We found that there is a strong positive correlation (R-squared = 0.92; p < 0.05) between known and inferred age of physiological stressors (pregnancy, menopause, incarceration, illness, relocation) (Fig. 2). The average inferred ages (over 10 counts) corresponding to the marked cementum bands, together with the known ages at childbirth, menopause and/or other possible physiologically demanding events are reported in Supplementary Table 1. The intercept value (y = 1.59) of the equation of the regression line reported in Fig. 2 (y = 0.94× + 1.59) indicates that there is a bias towards overestimating the age at event occurrence. This is likely caused by the constant (reflecting age at tooth emergence) added to our estimates. However, it is well known that cementum deposition actually begins before dental eruption is achieved (see Methods section for further discussion of the topic). The results summarized in Fig. 2 show a strong positive correlation between the actual age at reproduction and menopause and the inferred age of these events based on the corresponding cementum bands visible in histological thin sections imaged by polarized light (PL). The average error in the estimates of all known events (including reproductive, lifestyle and medical events) was 1.84 years with an average SD of 1.7.

All male individuals present a histomorphological change at an average age of 20 years, which does not correspond to any reported event. The single older male individual in the sample, PCM69, also presents a change in cementum histomorphology at the age of 50.8 years.

Female individual M21-08 died at age 25, without having had any children or having undergone menopause. Nevertheless, the cementum presents a distinct line inferred to correspond to 17.6 years of age. Her file reports that she moved away from her original rural environment to an urban one at the age of 18, determining a change in lifestyle and diet. Female individual PCF1 gave birth at the age of 36 and underwent menopause at the age of 38. We were not able to distinguish the two events as separate, but rather a single area of histomorphological change corresponding to the age of 39.7 years was detected. This individual also lactated for over a year, and never resumed regular menstrual cycling. In addition to the cementum marker at approximately 20 years, male individuals M02-02, M06-02, M09-02, M10-06 and M13-02 all present an additional marked area. While we know that all five of these individuals contracted severe systemic illnesses, we do not have information on the ages at which they occurred.

### Consistency across several teeth

**Figure 3:**
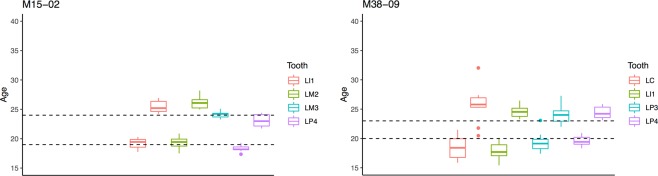
Boxplots showing the estimated ages at event occurrence for two individuals (M15-02 and M38-09). The boxes show the interquartile range (IQR); the line shows the median of the data; the whiskers add 1.5 times the IQR from the 75th percentile and subtract 1.5 times the IQR from the 25th percentile; the dots represent outliers. The transverse dotted black lines indicate the known age at event occurrence (two parturitions for M15-02; relocation and incarceration for M38-09).

For 9 of the 15 individuals we assessed the consistency of the detected signal across different teeth (Supplementary Table 2; Fig. 3). Some events were not detectable in all teeth: those occurring very early in life (either medical or physiological ones) were not present in the third molars, which are the last teeth to form. We performed an ANOVA to test whether the estimated age varied significantly between teeth (Supplementary Table 2). For seven out of nine individuals the differences in the estimates were statistically significant (p > 0.05) between teeth (Supplementary Table 2). This significant difference however, does not necessarily imply that the overall range estimated across the different teeth (average range = 2.5 years) does not provide meaningful information (Table 1). For the purposes of paleoanthropological (or even archeological and paleodemographic) questions even the widest range of our estimates (5.25 years) provides results that can enable us to tackle novel questions such as presence of a PRLS. We tested whether there was a correlation between a high precision of the measurements (a low SD) and a significant difference between the means of the different teeth, which could have been expected, but this was not the case. Furthermore, for each tooth type present we calculated the average error (Supplementary Table 3), defined as the absolute value of the difference between inferred age and known age at event occurrence. The results show that the single maxillary canine of our sample stands out as the tooth giving results with the highest error (5.5 years). Given that the sample size for this tooth type is one, we would not venture in making hypotheses regarding any correlation between tooth type and amount of error. The average error over all other teeth types is 1.44 years. We therefore suggest using several teeth from a given individual and averaging the results.

**Table 1:** Range of estimated values across several teeth of the same individual.

Individual	Teeth (n= )	Min age estimated (years)	Max age estimated (years)	Range
M21-08	2	17.65	19.94	2.29
M15-02	4	18.33	22.39	4.06
M15-02	5	23.01	26.01	3
M38-09	4	18.88	19.61	0.73
M38-09	4	24	25.7	1.7
M08-02	4	23.84	26.25	2.41
M08-02	4	31.89	35.6	3.71
M13-09	5	19.54	21.17	1.63
M13-09	5	29.88	32.62	2.74
M10-06	3	18.73	20.01	1.28
M10-06	3	23.56	24.81	1.25
M09-02	4	16.77	18.87	2.1
M09-02	5	20.74	22.82	2.08
M06-02	4	19.87	20.55	0.68
M06-02	4	28.35	33.6	5.25
M02-02	6	17.94	21.98	4.04
M02-02	6	32.18	35.85	3.67

For each event of each individual we report the number of teeth in which we assessed the signal (n= ); the minimum values inferred from a tooth of that induvial (in years); the maximum value inferred from a tooth of that individual (in years); the range (in years). The average range is 2.51 years. We therefore argue that the significant difference detected across the different teeth is determined be a high precision of the 10 measurements performed on each tooth. As the precision increases, so does the likelihood of detecting significant differences.

## Discussion

Our method assumes AEFC growth rate to be constant, as supported by other human^49^ and mammalian^42,48^ literature. The strong correlation (R-squared = 0.92) we found (Fig. 2) between known and inferred age at event occurrence supports the hypothesis that growth rate is constant in a given region of a given tooth of a given individual. Indeed, in a seminal study of 223 monoradicular teeth from known-age (11 to 76 years) individuals Zander and Hürzeler^49^ found that: increase in cementum thickness was near-linear; that mid-root cementum thickness best reflected age (according to the following regression y = 0.00359× + 0.0110); that the slope of the regression varied by tooth type. This finding does not contradict the research showing that cementum thickness is not always an accurate way to infer age^45^ because this would require cementum growth rate to be identical across different populations, which has not been tested by Zander and Hürzeler^49^, and for reference regressions to be available for each tooth type. It is therefore important to emphasize that we do not assume the species-wide rate of cementum formation to be fixed (which is at the base of age estimation) but rather that a single individual’s rate of cementum formation is constant. In addition, we did not find cementum thickness to be an indicator of the individual’s age, especially as differences in cementum thickness are noted along the longitudinal course of the root^43^ across both individuals and between teeth of the same individual (see Supplementary Table 6 and Supplementary Figures 1–9).

We did not observe yearly incremental bands in the dental cementum of the teeth sections imaged for this study. This result is surprising, as there is ample literature reporting the presence of yearly growth bands in the dental cementum of a variety of mammalian species^69,70^. We suspect that failure to discern yearly/seasonal incremental structures is the result of the infiltration process we followed during sample preparation. We are currently carrying out a study (unpublished data) comparing the effects of preparation protocol (dehydration and MMA embedding vs no dehydration and epoxy resin embedding) on the visibility of tooth cementum annulations (TCA). Our unpublished results show that adjacent cross sections of the same tooth, each of which underwent a different protocol, present a substantial difference in TCA visibility, with the MMA-embedded sections having a poor visibility. This finding is supported by the fact that some research attempting to validate TCA count for age estimation has had poor results when specimens were embedded in MMA^47^.

The bands of differential brightness we observed were only visible in polarized light, therefore reflecting changes in the birefringence of cementum. A difference in the refractive index of the material could be determined by either the organic (collagen) or inorganic (mainly HA) phase. Cool and colleagues^42^ compared normal, demineralized and decollagenized sections of the same teeth by imaging them in both electron and optical microscopy and found that the differences in birefringence are determined by the inorganic phase of cementum. The hypothesis that the contrasting bands observed are determined by changes in HA crystal size finds partial support in the body of evidence showing that in bone there are variations in HA crystal size in relation to ageing^71^, osteoporosis^72^ and menopause^73^. As this study did not detect yearly incremental bands it is possible that the yearly increments are determined by differences in mineral density whereas the physiologically-related bands are determined by the refractive index of the HA crystals.

All the males included in this study presented an unexpected change in cementum birefringence corresponding to approximately 20 years of age (with a range between 17.2 and 20.7). The timing of this change in cementum microstructure aligns closely with the timing of changes of free testosterone levels in males. It is known that free testosterone levels are high in neonates, but low during childhood^74^. They start increasing again at the beginning of puberty and reach a maximum at about 17 years of age, after which they slowly decline throughout the rest of the individual’s life^74^. Research has confirmed the function of testosterone in bone mass maintenance^75^ as this hormone is aromatized into estradiol^76^ inhibiting bone resorption^77,78^ by inducing osteoclast apoptosis^79^. Furthermore, recent research using cell proliferation assays has shown that cementoblast proliferation and cementum formation is promoted by 17β‐estradiol^80,81^. We therefore hypothesize that the histomorphological variation found in the dental cementum of males may be associated with changes in free testosterone. Further studies are needed to test this hypothesis.

A number of individuals presented changes in cementum microstructure not correlated with either reproductive status in females or regular changes in hormonal levels in males. We found bands closely corresponding to changes in lifestyle (in individuals M21-08, M38-09 and M08-02) or systemic illness (M08-02). These findings are consistent with those from the mammalian literature^13,31,65^ in which environmental stressors are correlated with alterations in cementum histomorphology. Furthermore, the five other individuals (M02-02, M06-02, M09-02, M10-06 and M13-02) presenting marked bands are reported to have been affected by a serious illness. However, the age at illness contraction is unknown so we cannot speculate whether or not there is a correlation with the changes seen in cementum.

Although our novel methodology has resulted in highly positive correlations between physiologically stressful events and changes in cementum, there are a number of limitations regarding its applicability to the fossil or archeological record. The destructiveness of the method can be overcome by implementing non-destructive phase-contrast synchrotron X-ray imaging^44^ which is sensitive not only to the density but also to the composition of an object^82^. The age and sex of the individual should be known. Information regarding sex is available in hominins preserving DNA. Moreover, recent research^83^ has shown that is it possible to determine the sex of human remains, from hundreds to thousands of years old, through the analysis of amelogenin peptides present in tooth enamel. The age of the individual at death/tooth extraction can be determined, if following the appropriate embedding protocol, through the count of yearly incremental cementum bands^44,46^. In the absence of medical and lifestyle records, it is impossible to determine what the cause of the change in cementum microstructure may be (illness, childbirth, relocation, etc.). This is an issue faced by all research dealing with stress markers in hard tissues. Dean and Elamin^16^ recognize the current impossibility to discern the cause of accentuated lines in the dentine of third molars (parturition events or illnesses); Kagerer and Grupe^38^ found the same results in cementum: renal diseases presented the same changes in cementum histomorphology as pregnancies. To address this issue our future work will include full elemental analysis of cementum, via laser ablation and mass spectrometry, with the aim of identifying event-specific changes in elemental composition. Finally, the applicability of this method to archeological or paleoanthropological material may be challenged by taphonomic changes. Studies looking at the yearly incremental bands have noted that taphonomic changes such as collagen leaching and apatite recrystallization can either mimic or hide true cementum yearly growth layers^40,84^. Although the impact of diagenetic factors has not been tested on specimens with known life history and age parameters, it is recommended that the samples used for the type of analysis we propose have minimal taphonomic alterations, as can be detected by macroscopic and mostly microscopic^84^ observation.

This study provides the first evidence in humans of histological markers corresponding not only to parturitions and menopause, but also illnesses and drastic changes in lifestyle. Furthermore, our results demonstrate that dental cementum constitutes a chronologically faithful biological archive of an individual, from which life history milestones, thus far not inferable from other mineralized tissues, can be detected and accurately timed. Furthermore, our results support the growing body of knowledge showing that mineralized tissues have a fundamental role participating in organismal physiology, endocrinology and mineral homeostasis.

## Methods

The research was carried out under the auspices of the College of Medicine Research and Ethics Committee (COMREC) Protocol Number: P.05/06/373 according to Malawi law regarding the Anatomy Act. All methods were carried out in accordance with relevant guidelines and regulations and all experimental protocols were approved by the NYU College of Dentistry. Informed consent was obtained from the next of kin of all the cadavers.

### Specimen preparation

The 47 male and female teeth used in this study (Supplementary Table 4) were processed and analyzed at the Hard Tissue Research Unit of the NYU College of Dentistry. All teeth were associated with the life history information of the individual from which they were extracted. The teeth derive from a cadaveric collection of Central African Malawians of Bantu origin. Some of the teeth (six specimens) were associated with only life-history information while the remaining 41 specimens were also associated with lifestyle information and medical history. The embedding protocol used for all the teeth was the *Hard Tissue Sample Processing and Embedding for LM and SEM* (Supplementary Table 5). The teeth were sectioned in their mid-longitudinal plane. The cured blocs were cut using a Beuhler IsoMet 1000 precision saw with diamond wafering blade. They were then polished with progressively finer grit sandpaper (600, 800 and 1200) on wet circular polisher and finally on wet cloth with Buehler MetaDi diamond suspension; then mounted on EXAKT microscope plastic slides using cyanoacrylate. Roots were sectioned to approximately 130 μm using the same Beuhler IsoMet 1000 precision saw with diamond wafering blade; they were then ground and polished to a thickness 100 +/−10 μm.

#### Imaging

We imaged the sections in the following way: for each slide PC made a preliminary assessment of the cementum using the Leica DMRXE at 1.3 and 2.6 micrometers per pixel magnification (Fig. 1). PC defined regions of interest (ROI) as areas of AEFC in which all the cementum layers visible were parallel to the cementum-dentine junction. This area was chosen to avoid imaging regions in which there was particular cell proliferation or where cementum deposition occurred in response to extrinsic factors such as disease, unusual masticatory loads, or other disrupting factors. Once we identified a ROI we proceeded to image it at higher magnification with different types of illumination: Bright Field (BF) and Differential Interference Contrast (DIC) using the Leica DM5000B and Polarized Light (PL) using the Leica DMRXE microscope. Digital micrographs were saved as tagged image file format (TIFF) files. Polarized light microscopy is useful for imaging the structure and composition of a number of organic and inorganic materials with characteristics related to their optically anisotropic character. Light has wave-like properties. The oscillations of the electromagnetic field are perpendicular to the direction in which the light propagates. Through the arrangement of two polarizing filters one vibration direction is isolated. The polarizer (first filter) is located between the light source and the specimen so that light can pass in only one direction of vibration (its axis of transmission). The analyzer (second filter) is instead located between the specimen and the observer and the direction of vibration permitted is orthogonal to that of the polarizer. Image contrast derives from the interaction of plane-polarized light with a birefringent specimen, thus producing two separate wave components, each one polarized in planes perpendicular to each other. In the absence of birefringent material in the light path, the analyzer blocks all the light coming from the polarizer (providing a black image). It is only in the presence of a birefringent material that interaction with the light exiting the polarizer occurs and the vibration of direction is changed so that light may pass through the analyzer and provide an image. Birefringent materials such as cementum display optically anisotropic characteristics when viewed in polarized light. Variations in brightness are determined by variations in light refraction by the material, which are direction-dependent. They are generated by differences in the orientation of the crystallographic axes present in the material. The birefringence patterns of cementum have been previously investigated experimentally^42^ through the comparative study of both demineralized and decollagenized sections and it was found that it was the negatively birefringent mineral component of the mineral (not the collagen) that dominates in image formation.

In all the samples yearly cementum annuli^46^ were either poorly visible or not discernible at all. Therefore, it was not possible to follow previously published methods^61,62^, in which parturitions were inferred by measuring the thickness of the yearly bands. Alternatively, we noted that in PL illumination the cementum displayed a small number (far fewer than the individual’s age subtracted from their age at dental eruption) of marked concentric layers presenting differential brightness.

### Estimation of age at event occurrence

**Figure 4:**
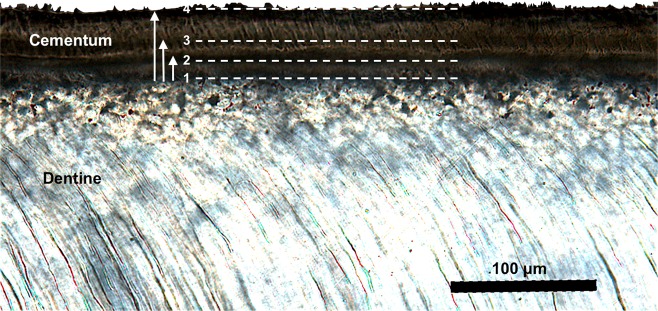
Micrograph obtained in polarized light (PL) of the cementum and a portion of dentine of the mandibular fourth premolar of individual M15-02. The line 1 corresponds to the cementum-dentine junction (CDJ). Lines 2 and 3 correspond to areas in which we observe changes in cementum birefringence. Total cementum thickness corresponds to the distance between lines 1 and 4. This distance corresponds to the age of the individual minus age at tooth completion. We therefore computed the yearly rate of cementum formation. We then used that rate to infer the amount of years elapsed between each subsequent cementum band: we therefore measured the distance between point 1 and 2 and between 1 and 3 and converted that distance into a number of years. Finally, we added the number of years corresponding to the amount of cementum measured to the age at tooth completion, thus inferring the age at event occurrence.

Using Adobe Photoshop we obtained the following measurements in the selected ROI: (i) total cementum thickness; and (ii) distance from the cementum-dentine junction (CDJ) to each subsequent visible cementum band (Fig. 4). Next, we established the ratio between the total cementum thickness and the number of years of cementum deposition. We estimated the number of years of cementum deposition as the individual’s age minus the average age at tooth eruption for the specific type of tooth (see section below)^21^. We then used the resulting proportional ratio to determine the number of years corresponding to each cementum band. Finally, we added that to the estimated age at full dental eruption^85^ and determined the age of the individual at each cementum band (Fig. 4). PC repeated the measurements 10 times for each specimen. Each measurement was performed blindly on randomized micrographs. A minimum of one week elapsed between each subsequent set of measurements.

#### Age at cementum secretion initiation

In this study (as with other studies aiming to assess age at death of individuals from tooth cementum annulations) there may be issues with determining the constant that is added to the number of years estimated through cementum analysis. Studies have conventionally added a value representing how long the individual lived before cementum secretion began. Tooth emergence is often used rather than tooth formation. Nevertheless, several definitions of “emergence” are present in the literature, such as gingival emergence and alveolar emergence. For a thorough review of the topic refer to Le Cabec and colleagues^44^. The present study follows the age estimates provided by AlQahtani and colleagues^85^ and the constant added is that of full dental eruption. By using those estimates we systematically overestimated the age at event occurrence. This is because cementum begins to form on the root before the root is complete^86^, hence cementum deposition begins earlier on the cervical than on the apical portion of the root. For an increased accuracy in future studies, we therefore suggest adding age constants that reflect the location on the root where the measurement is taken. For example, when measuring cementum in the cervical third of the root, age at ¼ to ½ of root completion should be used as the age at which cementum secretion begins. Several recent studies report ages at different stages of tooth formation^85^. Some of these studies^87,88^ report averages differentiated by sex and population and these variables should be taken into account, when available.

#### Cementum thickness measurement

For all the specimens used in the study we measured cementum thickness in two different regions of each tooth. To determine roughly homologous points in the roots of different teeth we followed the method established in enamel studies^89^, which involves subdividing the region of interest into deciles and measuring the thickness at given deciles. To calculate the length of the root surface we measured from the apex of the root to the cervix of the tooth. We then divided this length into ten equal segments (deciles). Using a Leica DMRXE microscope we collected micrographs at 1.3 micrometers per pixel magnification. Images were saved as TIFF image files and then scaled using FIJI ImageJ (NIH, Bethesda, MD, USA; https://imagej.nih.gov/ij/). Using the same software cementum thickness was measured on the scaled images at the second and fifth decile. We chose these deciles as the most extreme points of the root region (cervical half) expected to present AEFC. We excluded the first decile as non-pathological cementum damage can be common near the cervix^90^. Thickness measurements and summary plots are available in the Supplementary Information (Table 6 and Figs. 1–9).

## Supplementary information

Supplementary Information.

## Data Availability

Raw images and derived data supporting the findings of this study are available from the corresponding author (P.C.) on request.
