# Supplementary material for: Parturitions, menopause and other physiological stressors are recorded in dental cementum microstructure
Source: Sci Rep. 2020 Mar 25;10:5381. doi: 10.1038/s41598-020-62177-7 (PMC7096390; doi:10.1038/s41598-020-62177-7)
Supplement: Supplementary file 1 — Supplementary Information. [file 41598_2020_62177_MOESM1_ESM.pdf]

Parturitions, menopause and other physiological stressors are recorded in dental cementum microstructure

Paola Cerrito[1,2*], Shara E. Bailey[1,2], Bin Hu[3], Timothy G. Bromage[3]

[1]Department of Anthropology, New York University, New York, USA

[2]New York Consortium in Evolutionary Primatology, New York, USA

[3]Department of Biomaterials and Biomimetics, New York University College of Dentistry, New York, USA

*Corresponding author. Email: pc2294@nyu.edu

Supplementary Table 1. For each tooth with associated known physiologically impactful events we report the average estimated age over 10 counts; the standard deviation (SD) of the 10 measures; the known age at event occurrence; the difference between the estimate and the actual known value; the type of physiologically impactful event.

Specimen ID	Tooth	Average estimated age	SD	Known age at event occurrence	Difference	Event type
M21-08	LI1	17.65	0.77	18	0.35	Relocation
M21-08	LP4	19.94	0.92	18	1.94	Relocation
PCF56.2	UP3	34.7	2.87	30	4.7	Parturition
PCF56.2	UP3	50.47	1.33	50	0.47	Menopause
PCF56.1	UM3	30.95	3.99	30	0.95	Parturition
PCF56.1	UM3	46.79	1.8	50	3.21	Menopause
PCF68	UC	17.85	0.49	21	3.15	Parturition
PCF68	UC	26.06	0.86	24	2.06	Parturition
PCF68	UC	39.25	6.4	26	13.25	Parturition
PCF68	UC	54.37	7.98	51	3.37	Menopause
PCF64	UM3	32.96	1.72	31	1.96	Parturition
PCF64	UM3	48.23	1.07	50	1.77	Menopause
PCF1	LP3	39.7	2.53	38	1.7	Menopause
M15-02	LM3	24.12	0.6	24	0.12	Parturition
M15-02	LI1	19.2	0.82	19	0.2	Parturition
M15-02	LI1	25.48	0.96	24	1.48	Parturition
M15-02	LM1	22.39	0.4	19	3.39	Parturition
M15-02	LM1	25.64	0.82	24	1.64	Parturition
M15-02	LM2	19.32	0.99	19	0.32	Parturition
M15-02	LM2	26.01	0.95	24	2.01	Parturition
M15-02	LP4	18.33	0.5	19	0.67	Parturition
M15-02	LP4	23.01	1.02	24	0.99	Parturition
M38-09	LP4	19.61	0.83	20	0.39	Relocation
M38-09	LP4	24.42	0.97	23	1.42	Incarceration
M38-09	LC	18.44	1.9	20	1.56	Relocation
M38-09	LC	25.7	2.97	23	2.7	Incarceration
M38-09	LI1	17.88	1.29	20	2.12	Relocation

M38-09	LI1	24.59	0.97	23	1.59	Incarceration
M38-09	LP3	19.38	1.54	20	0.62	Relocation
M38-09	LP3	24	1.45	23	1	Incarceration
M08-02	LM3	24.95	1.06	25	0.05	Illness
M08-02	LM3	31.89	0.77	34	2.11	Incarceration
M08-02	UP3	26.25	2	25	1.25	Illness
M08-02	UP3	32.88	1.78	34	1.12	Incarceration
M08-02	LM1	23.84	1.44	25	1.16	Illness
M08-02	LM1	33.46	2.91	34	0.54	Incarceration
M08-02	LI1	25.9	1.59	25	0.9	Illness
M08-02	LI1	35.6	1.38	34	1.6	Incarceration

Supplementary Table 2. For each individual for which we had more than one tooth we performed an ANOVA to test whether the means of the inferred ages at event occurrence where significantly different across the several teeth of the same individual. In bold are highlighted the only three instances in which the means are not significantly different. For each specimen, tooth and event we also report the standard deviation of the set of 10 counts.

Specimen	Event	SD tooth 1	SD tooth 2	SD tooth 3	SD tooth 4	SD tooth 5	SD tooth 6	p-value
M02-02	1	0.84	1.92	2.16	1.37	0.73	2.20	1.23E-07
M02-02	2	0.91	1.28	1.76	1.28	1.34	2.05	8.08E-06
M06-02	1	1.57	0.39	1.16	0.50			0.416
M06-02	2	0.61	0.58	1.07	1.20			8.53E-14
M08-02	1	2.15	0.88	1.70				0.001
M08-02	2	1.68	2.11	1.52	1.12			0.009
M08-02	3	1.45	1.88	3.07	0.81			0.002
M09-02	1	0.48	0.49	0.63	1.69			1.37E-05
M09-02	2	0.36	0.55	0.48	1.09	0.48		4.39E-10
M10-06	1	0.77	0.94	0.97				0.007
M10-06	2	1.26	0.64	0.80				0.019
M13-02	1	1.58	1.48	1.10	1.53	1.79		**0.125**
M13-02	2	0.71	0.54	1.00	1.12	1.57	0.60	9.01E-07
M15-02	1	0.86	0.57	1.04				0.029
M15-02	2	1.01	1.08	1.00	0.63			6.51E-08
M21-08	1	0.82	0.97					2.09E-05
M38-09	1	1.36	2.01	1.63	0.88			**0.053**
M38-09	2	1.02	3.13	1.53	1.03			**0.226**

Supplementary Table 3. All the specimens present in Supplementary Table 2 are grouped by tooth type and the average error in age estimate per tooth type is calculated as the absolute value of the difference between known and inferred ages at event occurrence. Average error for upper canines (UC) would be 2.86 if eliminating the outlier with error of 13.25.

Specimen ID	Tooth	Difference (|known-inferred|)	Tooth type	Average difference per tooth type
M38-09	LC	1.56		
M38-09	LC	2.7		
			LC	2.13
M21-08	LI1	0.35		
M15-02	LI1	0.2		
M15-02	LI1	1.48		
M38-09	LI1	2.12		
M38-09	LI1	1.59		
M08-02	LI1	0.9		
M08-02	LI1	1.6		
			LI1	1.177
M15-02	LM1	3.39		
M15-02	LM1	1.64		
M08-02	LM1	1.16		
M08-02	LM1	0.54		
			LM1	1.682
M15-02	LM2	0.32		
M15-02	LM2	2.01		
			LM2	1.165
M15-02	LM3	0.12		
M08-02	LM3	0.05		
M08-02	LM3	2.11		
			LM3	0.76
PCF1	LP3	1.7		
M38-09	LP3	0.62		
M38-09	LP3	1		
			LP3	1.106
M21-08	LP4	1.94		
M15-02	LP4	0.67		
M15-02	LP4	0.99		
M38-09	LP4	0.39		
M38-09	LP4	1.42		
			LP4	1.082
PCF68	UC	3.15		
PCF68	UC	2.06		
PCF68	UC	13.25		
PCF68	UC	3.37		
			UC	5.457
PCF56.1	UM3	0.95		
PCF56.1	UM3	3.21		
PCF64	UM3	1.96		
PCF64	UM3	1.77		
			UM3	1.972
PCF56.2	UP3	4.7		
PCF56.2	UP3	0.47		
M08-02	UP3	1.25		
M08-02	UP3	1.12		
			UP3	1.885

Supplementary Table 4. List of specimens used in this study. For some specimens more than one tooth was analyzed. For each known event (1 through 4) the type of event and the age of the individual (in years) are reported.

Specimen	Tooth	Sex	Age	Event 1	Event 2	Event 3	Event 4
M02-02	LI1	M	40				
M02-02	LP3	M	40				
M02-02	UP4	M	40				
M02-02	LP4	M	40				
M02-02	LM1	M	40				
M02-02	LM2	M	40				
M06-02	LI1	M	38				
M06-02	LP3	M	38				
M06-02	LM1	M	38				
M06-02	LM2	M	38				
M08-02	LI1	M	42	25 Illness	34 Incarceration		
M08-02	UP3	M	42	25 Illness	34 Incarceration		
M08-02	LM3	M	42	25 Illness	34 Incarceration		
M08-02	LM1	M	42	25 Illness	34 Incarceration		
M09-02	LC	M	26				
M09-02	LP3	M	26				
M09-02	LP4	M	26				
M09-02	LM3	M	26				
M09-02	LM1	M	26				
M10-06	LP3	M	32				
M10-06	LP4	M	32				
M10-06	LM2	M	32				
M10-06	LP3	M	32				
M10-06	LP4	M	32				
M10-06	LM2	M	32				
M13-02	LI1	M	40				
M13-02	LC	M	40				
M13-02	LP3	M	40				
M13-02	LP4	M	40				
M13-02	LM3	M	40				
M13-02	LM2	M	40				
M38-09	LI1	M	42	20 Relocation	23 Incarceration		
M38-09	LC	M	42	20 Relocation	23 Incarceration		
M38-09	LP3	M	42	20 Relocation	23 Incarceration		
M38-09	LP4	M	42	20 Relocation	23 Incarceration		
PCM69	UP3	M	69				
M15-02	LI1	F	35	19 Parturition	24 Parturition		
M15-02	LP4	F	35	19 Parturition	24 Parturition		
M15-02	LM2	F	35	19 Parturition	24 Parturition		
M15-02	LM3	F	35	19 Parturition	24 Parturition		

M21-08	LI1	F	25	18 Relocation			
M21-08	LP4	F	25	18 Relocation			
PCF1	LP3	F	67	36 Parturition	38 Menopause		
PCF56.1	UM3	F	56	30 Parturition	50 Menopause		
PCF56.2	UP3	F	56	30 Parturition	50 Menopause		
PCF64	UM3	F	64	31 Parturition	50 Menopause		
PCF68	UC	F	68	21 Parturition	24 Parturition	26 Parturition	51 Menopause

Supplementary Table 5. Embedding protocol used for all the specimens present in the study.

Step	Material	Preparation
I. Dehydration		*Specimens can be stored at room temperature between changes; time left in each solution depends on size of samples*
1	70% Ethanol	One change, 12-48 hours, with vacuum
2	95% Ethanol	Two changes, 12-48 hours each, with vacuum
3	100% ethanol	Two changes, 12-48 hours each, with vacuum
II. Chemical preparation for embedding (cleaning, defatting)		*Specimens can be stored at room temperature between changes*
1	100% Methyl Salicylate	Two changes, 4-48 hours each, with vacuum
III. Infiltration		*Specimens in MMA-II and MMA-III must be stored at 4℃ – in the refrigerator – between changes, and brought to room temperature before each processing change step and before vacuum*
1	MMA-I	One change, 24 hours, with vacuum
2	MMA-II	One change, 36 hours, with vacuum
3	MMA-III	One change, 72 hours, with vacuum
IV. Embedding		
1	MMA-E	Embed sample into MMA-E solution (with vacuum, if viable, for 20 minutes). Cure sample at room temperature in the hood until mostly cured (minimum 24 hours, it will be hardened, but still 'sticky' when probed), then place samples under UV light at a distance of at least 0.75 meters for 24 hours followed by another 24 hours closer to the UV until cure is mostly complete. Finally samples can be stored in the oven at 37°C for 24 hours or exposure to UVA until cure is complete.

Supplementary Table 6. For each of the specimens present in the study: cementum thickness measurements (in micrometers) at the second and fifth deciles of the roots (first decile is at tooth cervix and tenth is at root apex).

Specimen ID	Tooth type	Age at apex closure	Age	Years of cementum	2nd decile cementum thickness (um)	2nd decile Cementum/year (um)	5th decile cementum thickness (um)	5th decile Cementum/year (um)
**M21-08**	LI1	6	25	19	21	1.105263	38	2
**M21-08**	LP4	12	25	13	26	2	41	3.153846
**M38-09**	LI1	6	42	36	29	0.805556	64	1.777778
**M38-09**	LC	9	42	33	53	1.606061	62	1.878788
**M38-09**	LP3	11	42	31	61	1.967742	95	3.064516
**M38-09**	LP4	12	42	30	32	1.066667	52	1.733333
**M15-02**	LI1	6	35	29	25	0.862069	46	1.586207
**M15-02**	LP4	12	35	23	21	0.913043	25	1.086957
**M15-02**	LM2	12	35	23	27	1.173913	34	1.478261
**M15-02**	LM3	18	35	17	21	1.235294	51	3
**M02-02**	LI1	6	40	34	22	0.647059	88	2.588235
**M02-02**	LP3	11	40	29	38	1.310345	68	2.344828
**M02-02**	UP4	14	40	26	69	2.653846	88	3.384615
**M02-02**	LP4	12	40	28	68	2.428571	74	2.642857
**M02-02**	LM1	6	40	34	70	2.058824	120	3.529412
**M02-02**	LM2	12	40	28	39	1.392857	101	3.607143
**M06-02**	LI1	6	38	32	41	1.28125	60	1.875
**M06-02**	LP3	11	38	27	44	1.62963	54	2
**M06-02**	LM1	6	38	32	41	1.28125	72	2.25
**M06-02**	LM2	12	38	26	40	1.538462	51	1.961538
**M08-02**	LI1	6	42	36	50	1.388889	45	1.25
**M08-02**	UP3	13	42	29	26	0.896552	39	1.344828
**M08-02**	LM3	18	42	24	31	1.291667	42	1.75
**M08-02**	LM1	6	42	36	53	1.472222	45	1.25
**M09-02**	LC	9	26	17	52	3.058824	80	4.705882
**M09-02**	LP3	11	26	15	28	1.866667	41	2.733333
**M09-02**	LP4	12	26	14	24	1.714286	26	1.857143
**M09-02**	LM1	6	26	20	15	0.75	35	1.75
**M09-02**	LM3	18	26	8	27	3.375	38	4.75
**M10-06**	LP3	11	32	21	21	1	43	2.047619
**M10-06**	LP4	12	32	20	12	0.6	51	2.55
**M10-06**	LM2	12	32	20	46	2.3	37	1.85
**M13-02**	LI1	6	40	34	48	1.411765	107	3.147059
**M13-02**	LC	9	40	31	29	0.935484	52	1.677419
**M13-02**	LP3	11	40	29	81	2.793103	*	

**M13-02**	LP4	12	40	28	76	2.714286	90	3.214286
**M13-02**	LM2	12	40	28	39	1.392857	72	2.571429
**M13-02**	LM3	18	40	22	40	1.818182	47	2.136364
**PFC1**	LP3	11	67	56	28	0.5	39	0.696429
**PCF56.1**	UM3	18	56	38	25	0.657895	37	0.973684
**PCF56.2**	UP3	11	56	45	30	0.666667	30	0.666667
**PCF64**	UM3	18	64	46	19	0.413043	32	0.695652
**PCF68**	UC	11	68	57	62	1.087719	76	1.333333
**PCM69**	UP3	13	69	56	43	0.767857	56	1

* For specimen 15-02 LP3 we were not able to collect cementum thickness at the fifth decile as it was not visible, likely due to a problem in sample preparation.

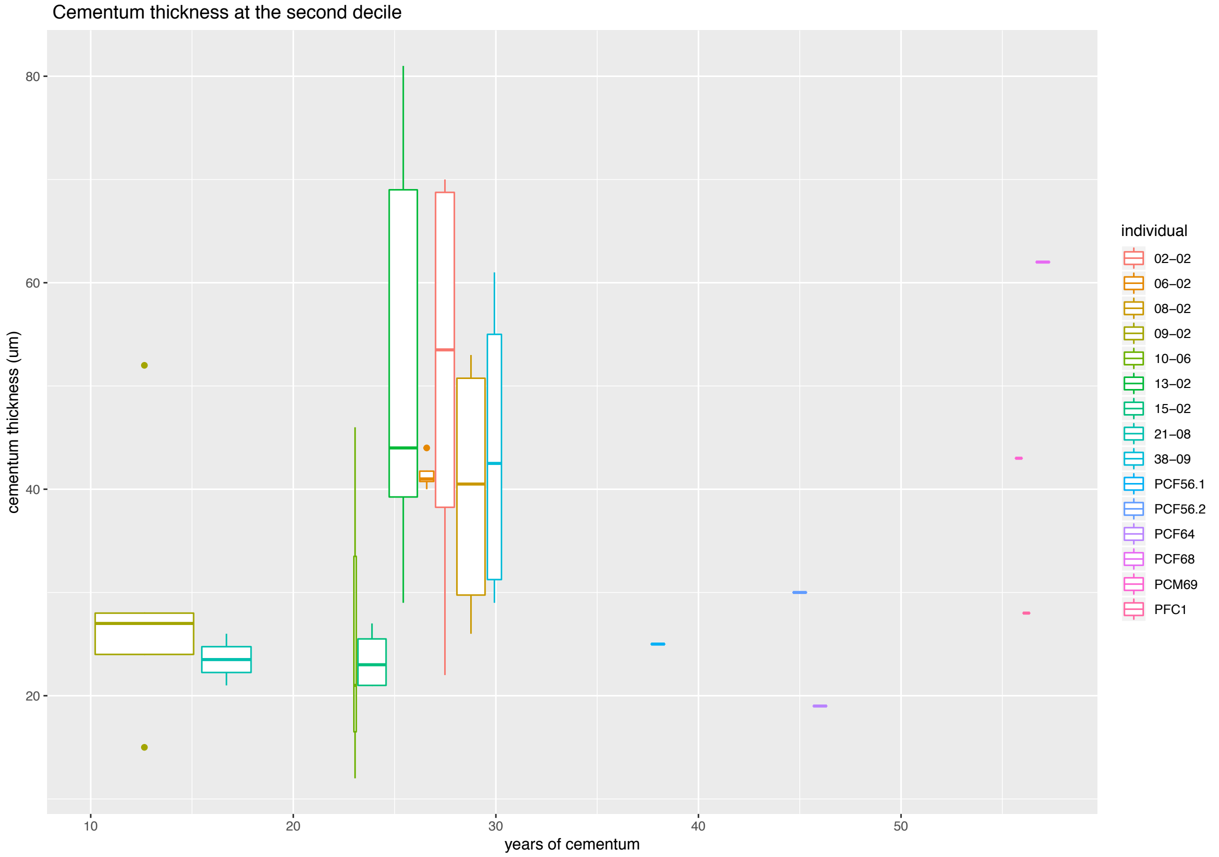

Supplementary Figure 1. Boxplot of cementum thickness (in micrometers), as measured on the fifth decile of the root, and years of cementum. Grouping (color) is by individuals.

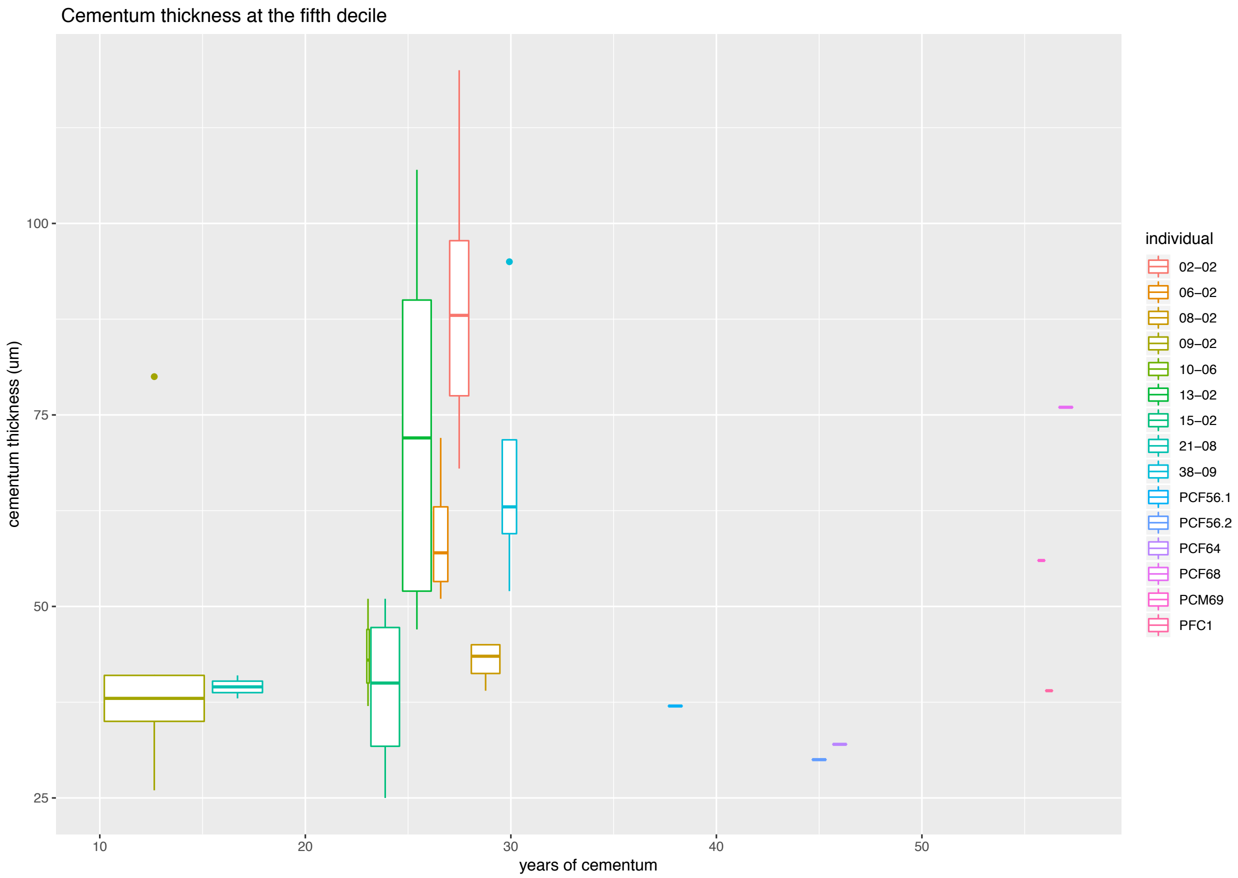

Supplementary Figure 2. Boxplot of cementum thickness (in micrometers), as measured at the fifth decile of the root, and years of cementum. Grouping (color) is by individuals.

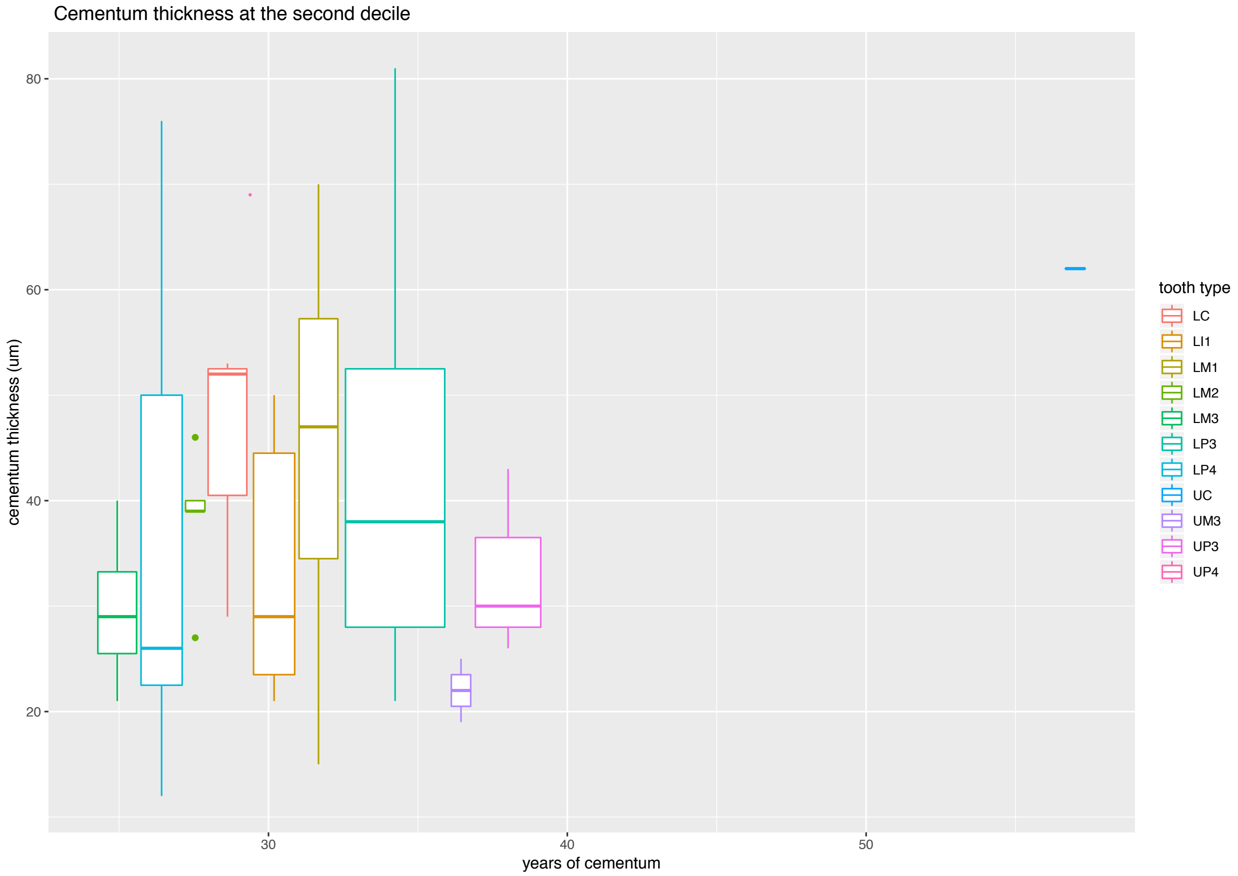

Supplementary Figure 3. Boxplot of cementum thickness (in micrometers), as measured at the second decile of the root, and years of cementum. Grouping (color) is by tooth type.

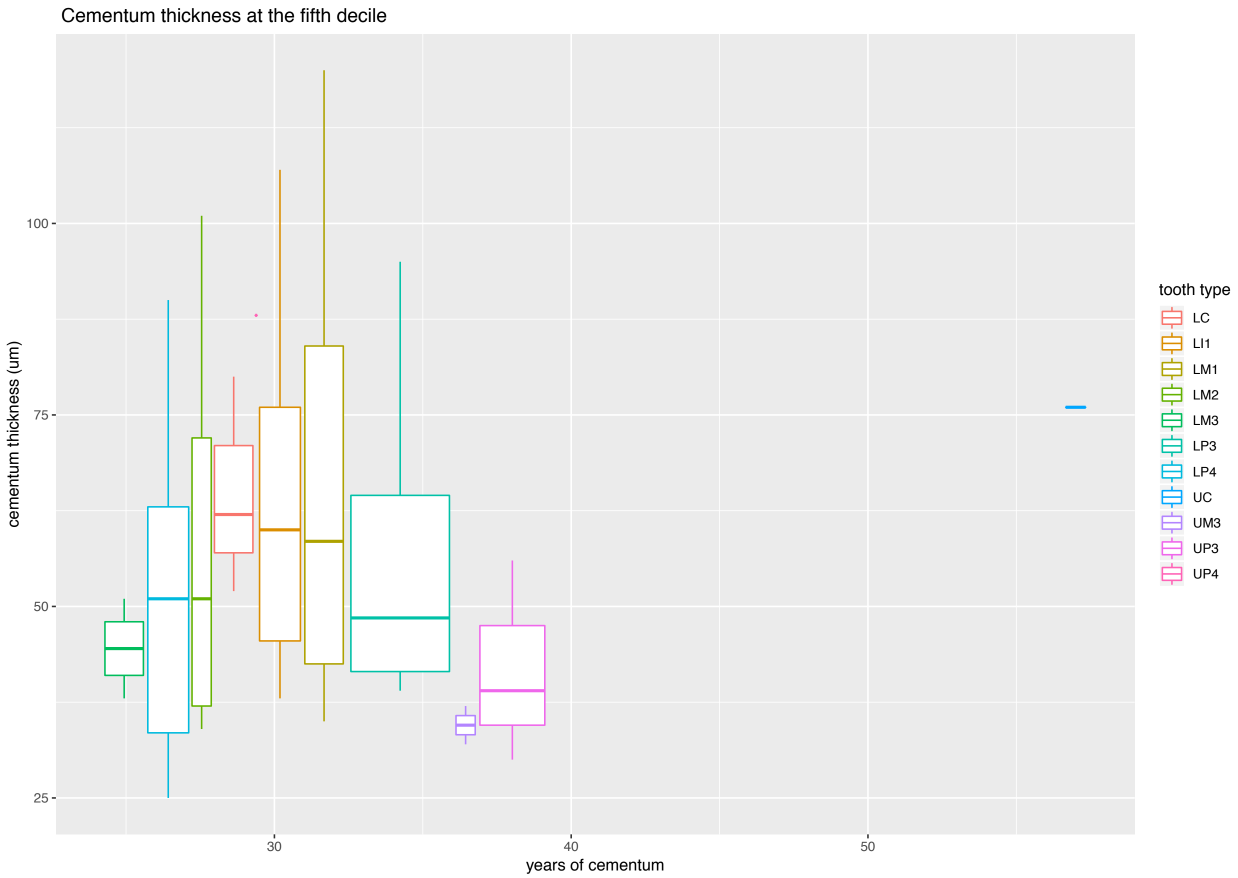

Supplementary Figure 4. Boxplot of cementum thickness (in micrometers), as measured at the fifth decile of the root, and years of cementum. Grouping (color) is by tooth type.

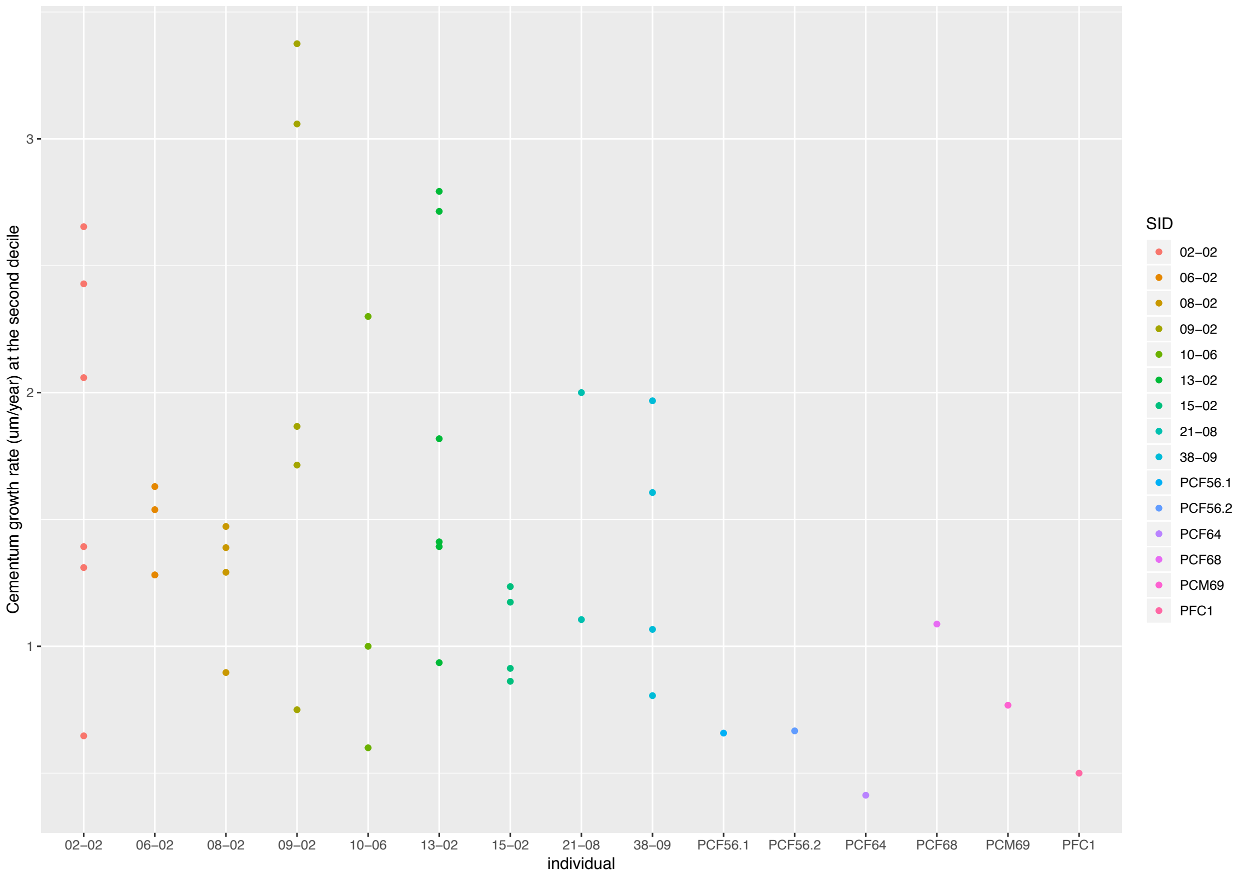

Supplementary Figure 5. Plot of cementum growth rate (in micrometers/year), as measured at the second decile of the root. Grouping (color) is by individual. Multiple dots for a single individuals indicate different teeth from the same individual. Between the different teeth of each individual there is ample range in growth rate.

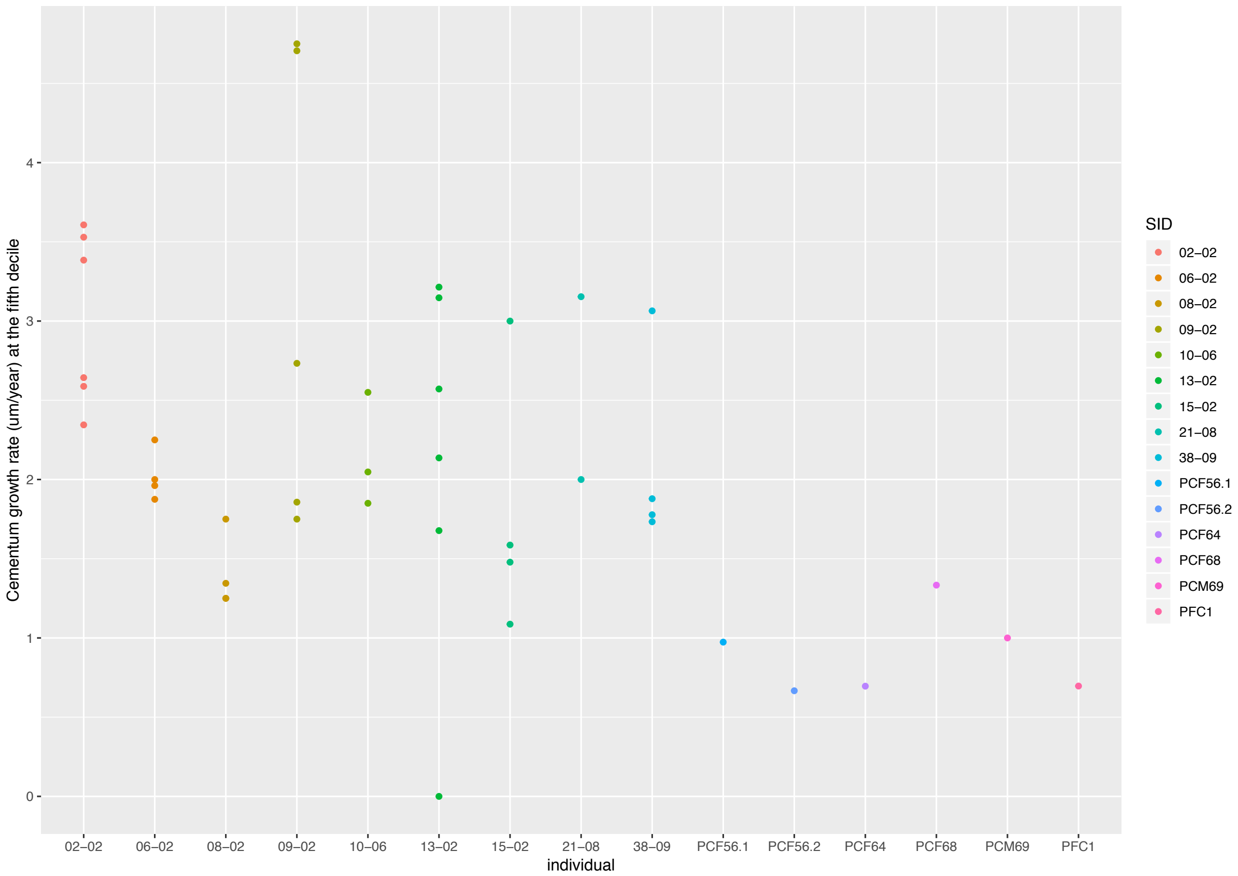

Supplementary Figure 6. Plot of cementum growth rate (in micrometers/year), as measured at the fifth decile of the root. Grouping (color) is by individual. Multiple dots for a single individuals indicate different teeth from the same individual. The value of zero for M13-02 is caused by absence of data. Between the different teeth of each individual there is ample range in growth rate.

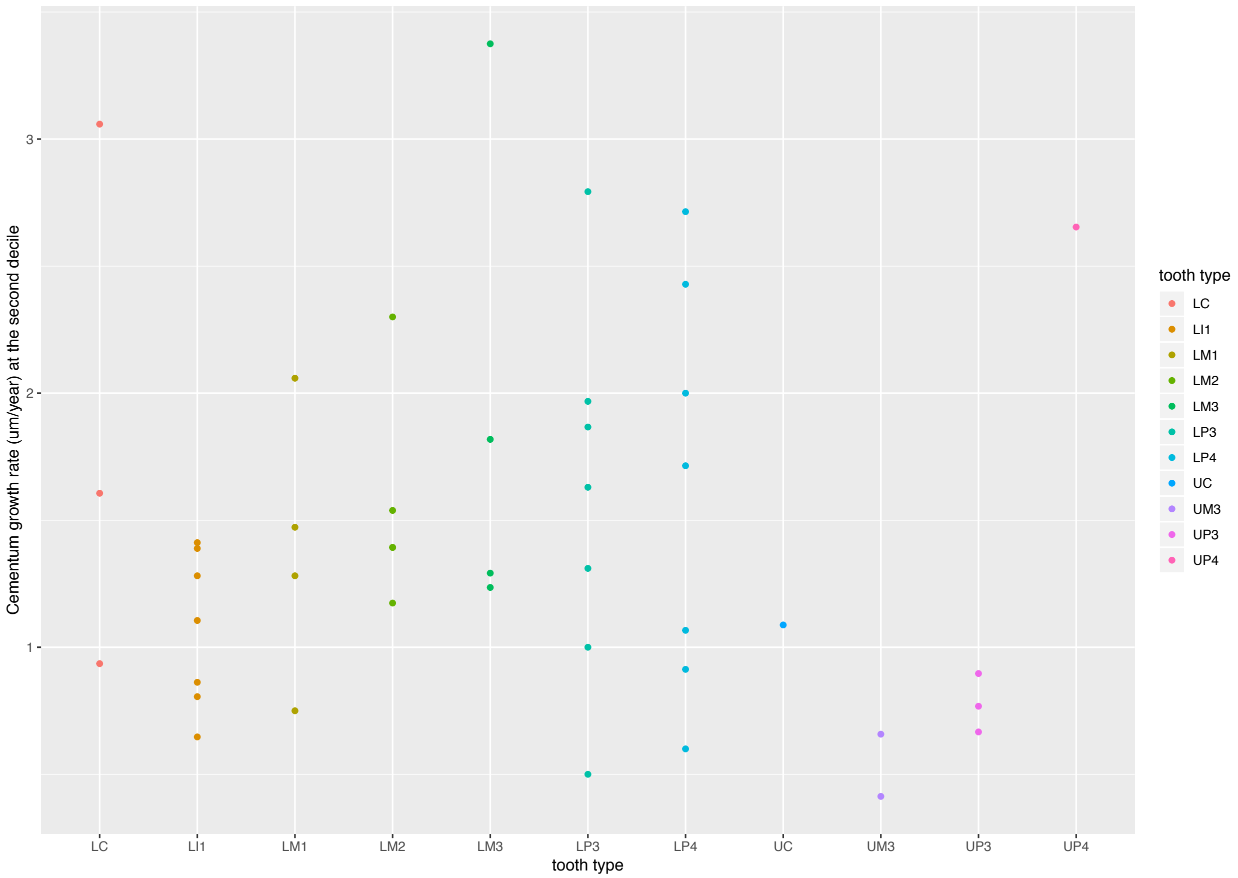

Supplementary Figure 7. Plot of cementum growth rate (in micrometers/year), as measured at the second decile of the root. Grouping (color) is by tooth type. Multiple dots for a same tooth type are each from different individuals. Amongst each tooth type there is ample range in growth rate.

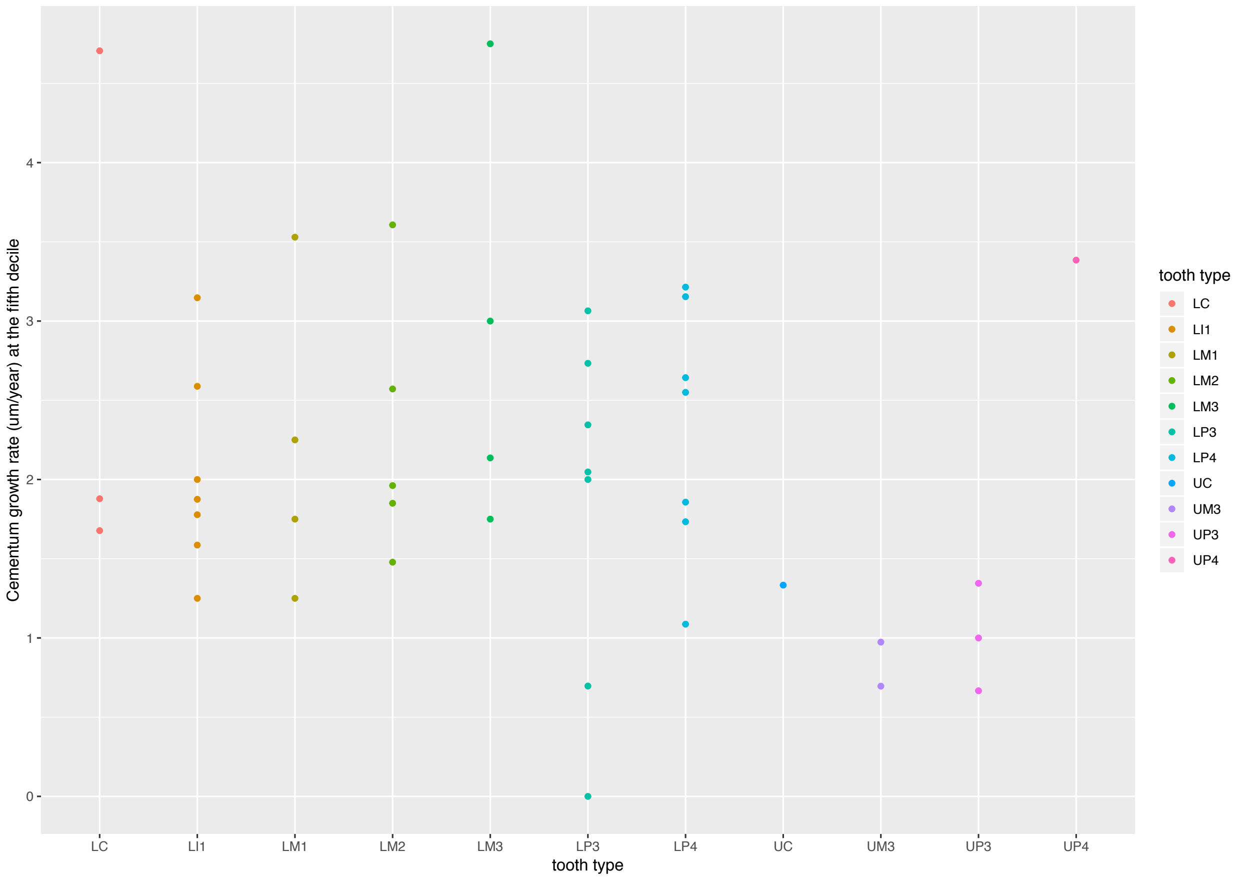

Supplementary Figure 8. Plot of cementum growth rate (in micrometers/year), as measured at the fifth decile of the root. Grouping (color) is by tooth type. Multiple dots for a same tooth type are each from different individuals. The value of zero for M13-02 is caused by absence of data. Amongst each tooth type there is ample range in growth rate.

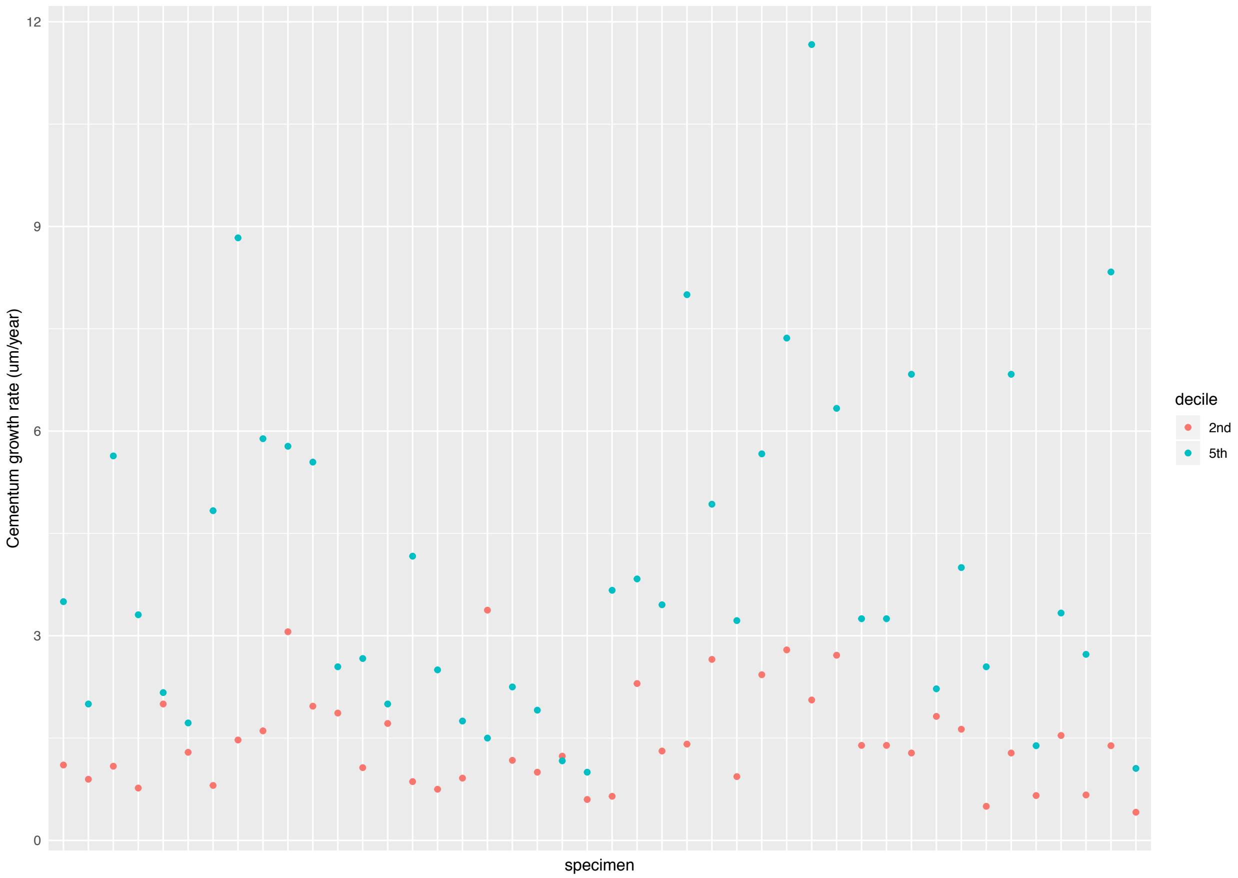

Supplementary Figure 9. Plot of cementum growth rate (in micrometers/year), as measured at the second (blue) and fifth (red) deciles of the root, for each of the specimens used in the study (x axis). Each specimen has two data points. Very few specimens have coinciding values for the second and fifth deciles. This indicates differences in growth rate along the length of the root.
